# Integrated Bioinformatics and Clinical Correlation Analysis of Key Genes, Pathways, and Potential Therapeutic Agents Related to Diabetic Nephropathy

**DOI:** 10.1155/2022/9204201

**Published:** 2022-05-21

**Authors:** Shengnan Chen, Lei Chen, Hongli Jiang

**Affiliations:** Department of Blood Purification, Kidney Hospital, The First Affiliated Hospital of Xi'an Jiaotong University, Xi'an, Shaanxi 710061, China

## Abstract

**Background:**

Diabetic nephropathy (DN) is a common microvascular complication of diabetes and a major cause of end-stage renal disease, resulting in a substantial socioeconomic burden around the world. Some unknown biomarkers, mechanisms, and potential novel agents regarding DN are yet to be identified.

**Methods:**

GSE30528 and GSE1009 were downloaded as training datasets to identify differentially expressed genes (DEGs) of DN. Common DEGs were selected for further analysis. Gene Ontology and Kyoto Encyclopedia of Genes and Genomes (KEGG) analyses of DEGs were performed to explore molecular mechanisms and pathways. Protein-protein interaction (PPI) network of DEGs was used to identify the top 10 hub genes of DN. Expression profiles of the hub genes were validated in GSE96804 and GSE47183 datasets. The clinical correlation analyses were conducted to confirm the association between key genes and clinical characteristics in the Nephroseq v5 database. The Drug Gene Interaction Database was used to predict potential targeted drugs.

**Results:**

345 and 1228 DEGs were identified in GSE30528 and GSE1009, respectively; and 120 common DEGs were found. The biological process of DEGs was significantly enriched in kidney development. PI3K-Akt signaling pathway, focal adhesion, complement and coagulation cascades were significantly enriched KEGG pathways. The identified top10 hub genes were VEGFA, NPHS1, WT1, TJP1, CTGF, FYN, SYNPO, PODXL, TNNT2, and BMP2. VEGFA, NPHS1, WT1, CTGF, SYNPO, PODXL, and TNNT2 were significantly downregulated in DN. VEGFA, NPHS1, WT1, CTGF, SYNPO, and PODXL were positively correlated with glomerular filtration rate. The targeted drugs or molecular compounds were enalapril, sildenafil, and fenofibrate target for VEGFA; losartan target for NPHS1; halofuginone, deferoxamine, curcumin, and sirolimus target for WT1; and purpurogallin target for TNNT2.

**Conclusions:**

VEGFA, NPHS1, WT1, CTGF, SYNPO, and PODXL are promising biomarkers for diagnosing and evaluating the progression of DN. The drug-gene interaction analyses provide a list of candidate drugs for the precise treatment of DN.

## 1. Introduction

Diabetic nephropathy (DN) is one of the most common microvascular complications of diabetes [1] that approximately 30% to 40% of diabetes patients may develop DN [2]. Currently, the incidence of diabetes mellitus is increasing year by year worldwide [3, 4]. With the rising prevalence of diabetes mellitus, DN has become the leading cause of chronic kidney disease (CKD) and end-stage renal disease (ESRD) in China [5, 6]. Meanwhile, patients with DN have a higher risk of cardiovascular disease (CVD) [4] which imposes a heavy health and economic burden on individuals, families, and society. Despite abundant studies and efforts have been made to understand and manage DN, there is still a large residual risk for DN patients. Current clinical practice guidelines still fall short of strategies to halt the progression of DN [7] since it is a complex metabolic disorder which involves many mechanisms and pathways.

Therefore, some unknown mechanisms and corresponding potential novel agents are yet to be discovered for the better control of DN. In recent years, some microarray data analyses on DN have been carried out [8, 9] and provide comprehensive evidence that the genetic background is involved in the development of DN [10]. Therefore, there are likely many key genes that can be used as biomarkers for the identification of DN [11]. Human gene expression profiling can be useful in providing potential pathophysiological mechanisms and identifying promising biomarkers and potential novel drugs for DN [12]. With the rapid development of high throughput sequencing technologies, integrated bioinformatics analysis has emerged as a promising approach to further explore human gene expression profiling. Thus, with the aim to provide new insights to uncover potential key genes and pathways associated with DN, we used the integrated bioinformatics methods to investigate the human kidney biopsy tissue gene expression profiles.

In the present study, we identified differentially expressed genes (DEGs) of DN by analyzing two RNA expression profiles (training datasets) that were downloaded from the Gene Expression Omnibus (GEO) database. Subsequently, common DEGs of training datasets were identified for further analyses. Gene Ontology (GO) and Kyoto Encyclopedia of Genes and Genomes (KEGG) enrichment analyses were performed to explore potential molecular mechanisms and pathways of DN. We also constructed the protein-protein interaction (PPI) network and identified the top 10 hub genes of DN. Expression profiles of the hub genes were validated in the external validation datasets to prove the reliability of our results. And the clinical correlation analyses were performed to confirm the clinical significance of the screened key genes. Finally, we predicted the potential drugs associated with key genes of DN. It is anticipated that our study will shed light on the underlying molecular mechanisms and provide several reliable biological markers for diagnosing and monitoring DN. Meanwhile, the results would provide some theoretical basis for exploring novel targeted agents to delay the progression of DN or improve the prognosis of DN patients.

## 2. Materials and Methods

### 2.1. Microarray Data Resources and Collection

The gene expression datasets analyzed in this study were obtained from the National Center of Biotechnology Information (NCBI) GEO database (https://www.ncbi.nlm.nih.gov/geo). The keywords “diabetic nephropathy” or “diabetic kidney disease” were used to search on the GEO database. After a careful review, two total RNA expression profiling datasets (GSE30528 and GSE1009) containing renal glomerular tissue samples of DN and normal population were selected as training datasets, and their series matrix file and platform annotation information were downloaded for further analyses.

### 2.2. Identification of DEGs

The downloaded platform and series matrix files were converted using the R software (version 4.1.1). The ID corresponding to the probe name was converted into an international standard name for genes (gene symbol). Identification of DEGs between DN and normal controls was performed using the limma package in the Bioconductor package (http://www.bioconductor.org/). Genes with an adjusted *P* value of < 0.05 and the log2 fold change (FC) value ≥ 1 or ≤ −1 (|log2 FC| ≥ 1) were considered as DEGs. In addition, Volcano plots for DEGs were created via ggplot2 package (version 2.2.1). Venn plots were used to intersect the two datasets (GSE30528 and GSE1009) to obtain the common DEGs.

### 2.3. GO and KEGG Pathway Enrichment Analyses of DEGs

To get a better understanding of the function of DEGs, DAVID (https://david.ncifcrf.gov/) and R software were used to conduct KEGG pathway enrichment analyses of common DEGs. GO analyses consisting of biological process (BP), cellular component (CC), and molecular function (MF) were also performed. The top 5 statistically significant enriched BP, CC, and MF terms and all enriched KEGG pathways were recorded.

### 2.4. Construction of PPI Network and Identification of Hub Genes

PPI network was established via the online tool STRING (http://string-db.org/) by importing the common DEGs of GSE30528 and GSE1009, and analytic results were downloaded with a confidence score > 0.40. Subsequently, the PPI network was visualized by Cytoscape software (version 3.6.0). CytoHubba, a plugin in Cytoscape, was used to calculate the degree of each protein node, and the top ten genes were identified as hub genes.

### 2.5. Expression Profiles of Hub Genes in External Validation Datasets

To further prove the reliability of our results, we compared the expression levels of the identified hub genes of DN in RNA expression profiling datasets (GSE96804 and GSE47183) from GEO database for external validation. The samples of these two datasets were glomeruli from DN patients and the normal portion of tumor nephrectomies (control group). Unpaired Student's *t*-tests were used to compare normally distributed data with homogeneous variance between two groups while nonnormally distributed data were compared by Mann–Whitney *U* test. All tests were two-tailed, a *P* value < 0.05 was considered as statistically significant. All data were analyzed and graphed by GraphPad Prism version 9.0.

### 2.6. Association between the Hub Genes and Clinical Characteristics

Nephroseq v5 online database (http://v5.nephroseq.org) was used to validate the correlation between the expression level of hub genes and clinical indicators. The correlation analysis was conducted by Pearson's correlation coefficients. *P* < 0.05 was considered as statistically significant.

### 2.7. Identification of the Potential Drugs

The Drug Gene Interaction Database (DGIDB) [13] online database (https://www.dgidb.org) was used to predict the potential targeted drugs that interacted with the verified hub genes, and then the Cytoscape software was used to construct drug-gene interaction network.

## 3. Results

### 3.1. Evaluation and Characteristics of the Data

**Figure 1:**
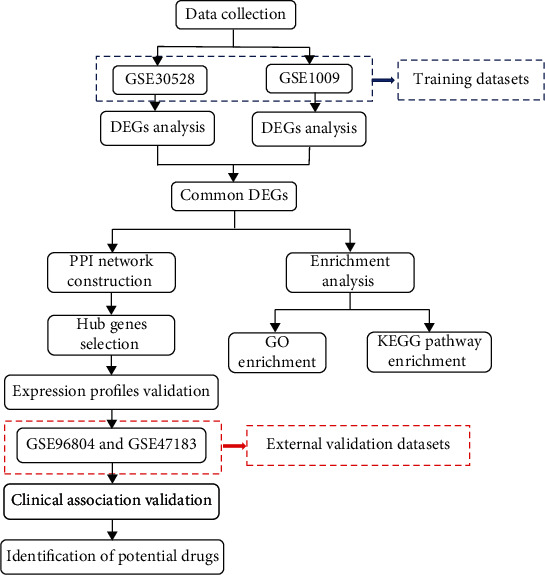
Flowchart of the study. GSE30528 and GSE1009 were defined as training datasets to screen DEGs, and the intersection was taken as common DEGs to perform functional enrichment analysis. The common DEGs were used to construct PPI network and identify the top 10 hub genes of DN. GSE96804 and GSE47183 were defined as external validation datasets to verify the gene expression profiles of the screened hub genes. The clinical correlation analysis and drug-gene interaction analysis were performed to validate the clinical significance and potential targeted drugs of hub genes associated with DN.

The platform for GSE30528 is GPL571 platform ([HG-U133A_2] Affymetrix Human Genome U133A 2.0 Array) which includes 13 healthy control's and 9 DN patients' renal glomeruli samples. The platform for GSE1009 is GPL8300 platform ([HG_U95Av2] Affymetrix Human Genome U95 Version 2 Array) which includes 3 healthy control's and 3 DN patients' renal glomeruli samples.  Figure 1 shows the flowchart of our study.

### 3.2. Identification of DEGs in DN

**Figure 2:**
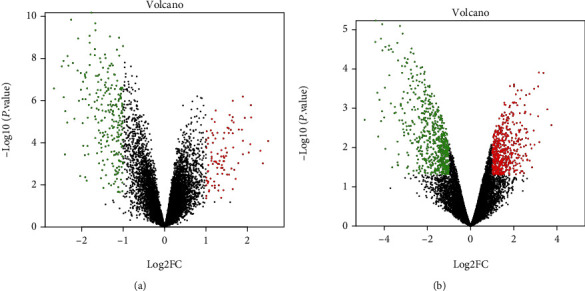
Volcano plots of DEGs in the DN group vs. normal control. (a) GSE30528, (b) GSE1009. Each symbol represents a different gene. The black dots represent the genes expressed without significant differences. The red color of the symbols represents upregulated genes, whereas points in green represent downregulated genes.

Two gene expression profiles (GSE30528 and GSE1009) were analyzed in this study. A total of 345 DEGs were identified from GSE30528 based on the defined criteria, 99 were upregulated genes and 246 were downregulated genes in the DN group, and these DEGs are shown in the volcano plots (Figure 2(a)). 1228 DEGs were screened from GSE1009, including 579 upregulated genes and 649 downregulated genes in the DN group (Figure 2(b)).

### 3.3. Screening for the Common DGEs

**Figure 3:**
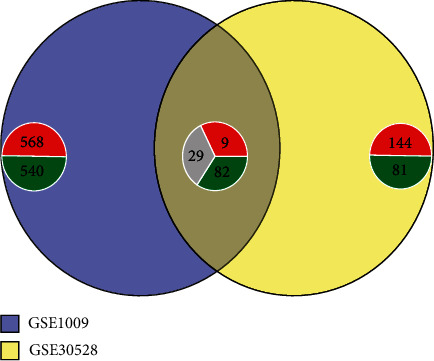
Common DGEs of GSE30528 and GSE1009. Red represents upregulated genes, green represents downregulated genes, and gray indicates genes changed in the opposite direction between GSE1009 and GSE30528.

Common DGEs were analyzed by comparing GSE30528-DEGs with GSE1009-DEGs. 120 common DEGs were identified, including 9 common upregulated DEGs (CCL19, FCER1A, IL7R, LDB2, MRC1, SERPINF1, TDO2, TRBC1, and TSPAN1); 82 common downregulated DEGs such as BCAR3, CTGF, DPP6, NPHS1, PODXL, PLA2R1, SYNPO, TGFBR3, TNNT2, VEGFA, WT1, ZNF185, and ZNF423; and 29 changed DEGs (Figure 3).

### 3.4. Enrichment Analysis of Common DEGs

To identify relevant pathways and functions of common DEGs, GO functional enrichment analysis and KEGG pathway enrichment analysis were performed for these 120 common DEGs.

**Figure 4:**
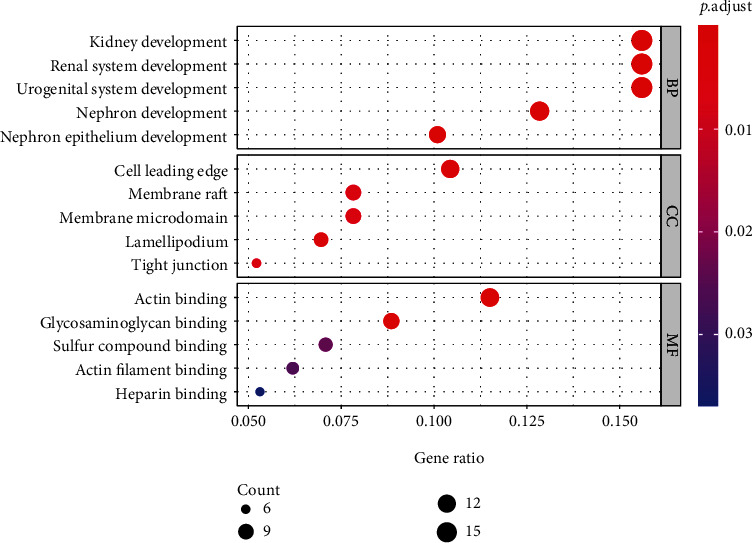
GO enrichment analysis of common DEGs. The *x*-axis label represents the gene ratio (the number of genes enriched in one GO term divided by the total number of genes used for enrichment analysis), and the *y*-axis label represents GO terms. The color of the node is displayed in a gradient from red to blue according to the ascending order of the adjusted *P* value.

**Figure 5:**
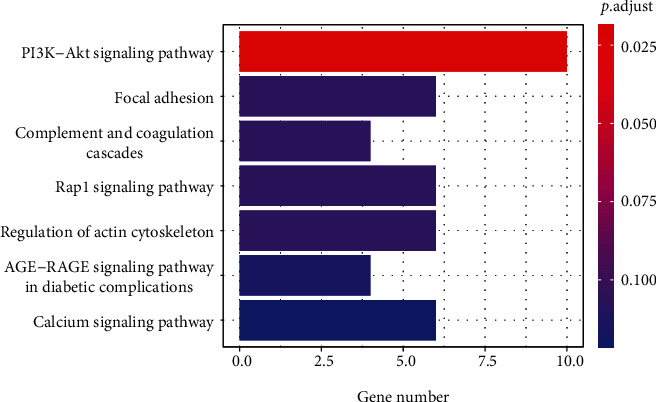
KEGG pathway enrichment analysis of common DEGs. The *x*-axis label represents the gene number, and the *y*-axis label represents signaling pathways. The color of the bar is displayed in a gradient from red to blue according to the ascending order of the adjusted *P* value.

The results of the GO enrichment analysis (Figure 4) indicated that common DEGs were significantly enriched in kidney development, renal system development, urogenital system development, nephron development, and nephron epithelium development in BP. For CC terms, DEGs were significantly enriched in cell leading edge, membrane raft, membrane microdomain, lamellipodium, and tight junction. For MF, DEGs were significantly enriched in actin binding, glycosaminoglycan binding, sulfur compound binding, actin filament binding, and heparin binding. As revealed from the KEGG enrichment analysis, the signaling pathways were mainly enriched in the PI3K-Akt signaling pathway, focal adhesion, complement and coagulation cascades, Rap1 signaling pathway, regulation of actin cytoskeleton, AGE-RAGE signaling pathway in diabetic complications, and calcium signaling pathway (Figure 5).

### 3.5. PPI Network Construction and Hub Gene Identification

**Figure 6:**
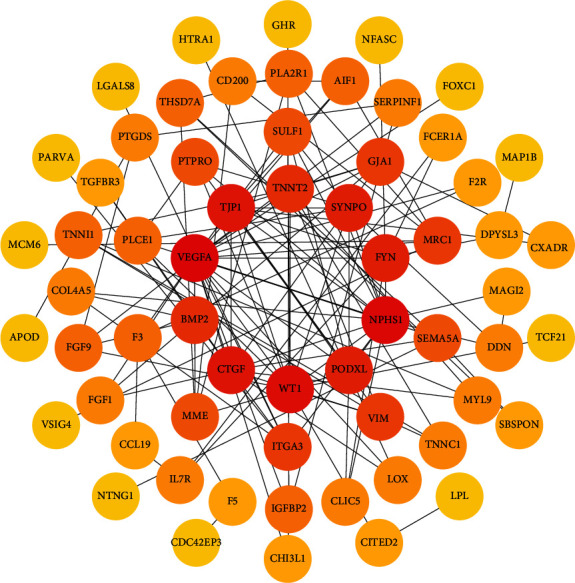
The PPI network of common DEGs and top 10 hub genes of DN. The top 10 hub genes were represented in the central of the network. The color of the nodes reflects the degree of connectivity (red color represents a higher degree, and yellow color represents a lower degree).

The120 common DEGs were uploaded to the STRING online database to acquire the information on PPI networks. A total of 118 nodes and 133 edges were covered in the network. The top10 hub genes were screened by Cytoscape; they were VEGFA, NPHS1, WT1, TJP1, CTGF, FYN, SYNPO, PODXL, TNNT2, and BMP2 (Figure 6).

### 3.6. Expression Profiles of Hub Genes in External Validation Datasets

**Figure 7:**
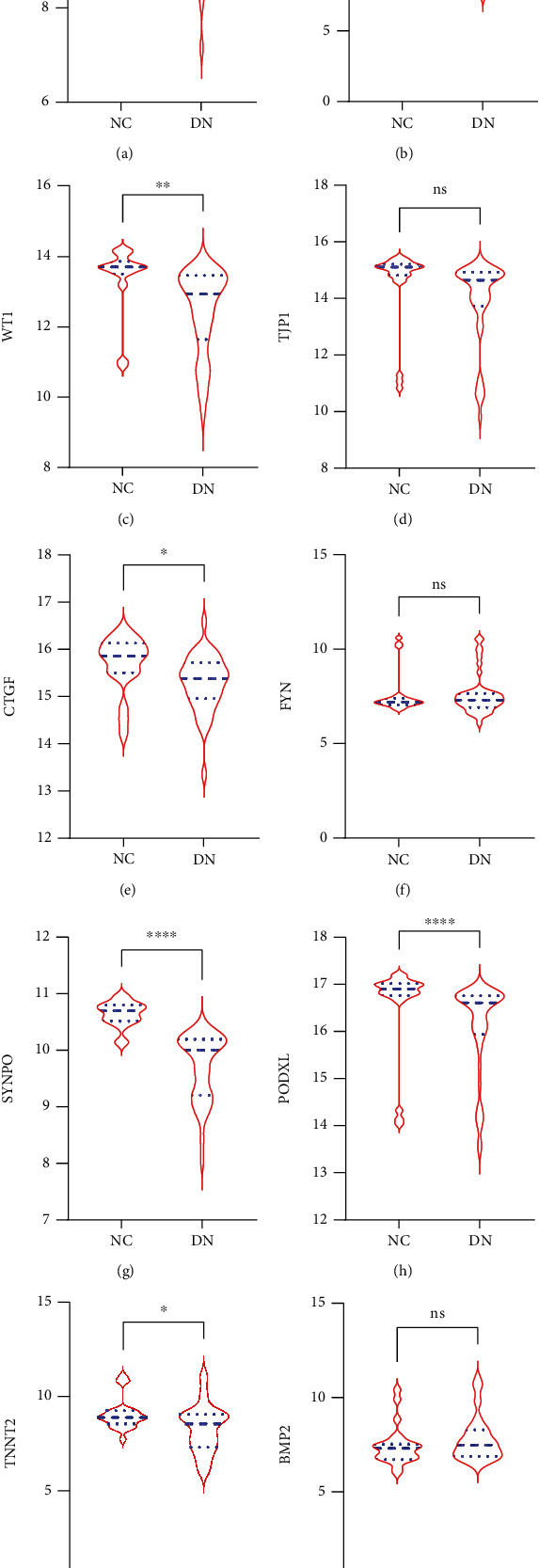
Expression profiles of hub genes in DN compared with NC in the GSE96804 and GSE47183 datasets. The expression level of (a) VEGFA, (b) NPHS1, (c) WT1, (d) TJP1, (e) CTGF, (f) FYN, (g) SYNPO, (h) PODXL, (i) TNNT2, and (j) BMP2. DN: diabetic nephropathy; NC: normal control. ∗∗∗∗*P* < 0.0001, ∗∗∗*P* < 0.001, ∗∗*P* < 0.01, ∗*P* < 0.05, ns: *P* > 0.05.

We analyzed the expression profiles of the hub genes in GSE96804 which including 41 samples of DN and 20 samples of control group and GSE47183 which include 7 DN and 3 control samples. All of the hub genes can be analyzed in validation datasets. The results in GSE96804 and GSE47183 showed that there was no significant difference in the expression level of TJP1, FYN, and BMP2 between DN and control group. For the reason that the expression level of TJP1 and BMP2 was also inconsistent in GSE1009 and GSE30528, the significance of TJP1 and BMP2 in DN cannot be verified. Although the expression level of FYN in DN group was significantly downregulated in GSE1009 and GSE30528, there was no significant difference of FYN level in GSE96804 and GSE47183 datasets. So, the FYN expression profile cannot be verified in external validation datasets. Except for these three genes, all the other hub genes can be verified in external validation datasets and consistent with the results of training datasets that VEGFA, NPHS1, WT1, CTGF, SYNPO, PODXL, and TNNT2 were significantly downregulated in the DN renal glomeruli tissues compared with control samples (Figure 7).

### 3.7. Validation of Association between Verified Hub Genes and Clinical Characteristics

**Figure 8:**
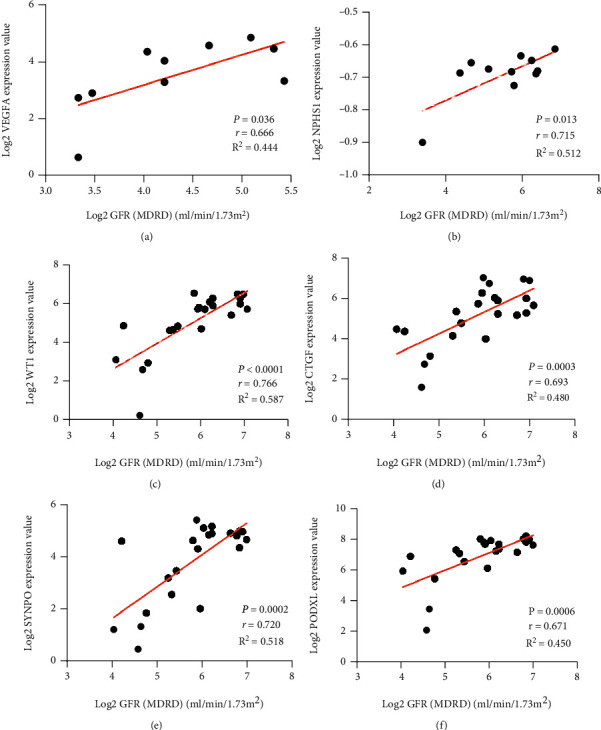
Correlation analysis between the expression of hub genes and renal function. The expression of (a) VEGFA, (b) NPHS1, (c) WT1, (d) CTGF, (e) SYNPO, and (f) PODXL were positively correlated with GFR.

The association between the verified 7 hub genes (VEGFA, NPHS1, WT1, CTGF, SYNPO, PODXL, and TNNT2) and renal function (glomerular filtration rate: GFR) was performed by Pearson's correlation analysis. Except that the expression level of TNNT2 in DN patients was not recorded in Nephroseq database, the expression level of VEGFA and NPHS1 mRNA in glomeruli of DN was positively correlated with GFR. And the expression level of WT1, CTGF, SYNPO, and PODXL was positively correlated with GFR in DN and the control group. The expression of VEGFA, NPHS1, WT1, CTGF, SYNPO, and PODXL decreased with the decrease of GFR (Figure 8).

### 3.8. Identification of the Potential Drugs

**Figure 9:**
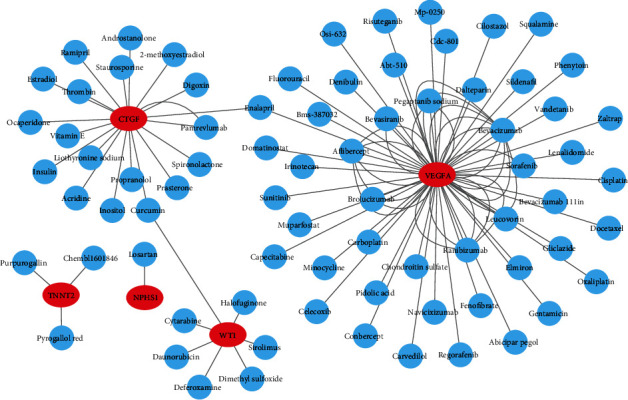
The drug-gene interaction network. The red oval nodes represent the genes, and the blue circle nodes mean the drugs.

VEGFA, NPHS1, WT1, CTGF, SYNPO, PODXL, and TNNT2 were verified DEGs both in the training datasets (GSE30528 and GSE1009) and external validation datasets (GSE96804 and GSE47183). Therefore, these seven genes were searched in the DGIDB database to identify targeted drugs. As shown in the drug-gene interaction network (Figure 9), drugs such as enalapril, ranibizumab, pegaptanib sodium, bevasiranib, aflibercept, risuteganib, and brolucizumab could regulate the expression of VEGFA. Losartan could regulate the expression of NPHS1. Dimethyl sulfoxide, halofuginone, deferoxamine, and curcumin could regulate the expression of WT1. Pamrevlumab, acridine, ramipril, enalapril, vitamin E, and 2-methoxyestradiol could regulate the expression of CTGF. Purpurogallin, pyrogallol red, and chembl1601846 could regulate the expression of TNNT2.

## 4. Discussion

DN is characterized by glomerulosclerosis, thickening of the glomerular basement membrane, podocyte loss, and tubulointerstitial fibrosis, which ultimately result in progressive albuminuria and reduction in GFR [14]. This hints that glomerular lesions play a key role in the development of DN and the biopsy of renal tissue is the gold standard for diagnosing DN [15]. Therefore, biomarkers from renal tissue are of great significance for the diagnosis and evaluation of DN. Hence, we analyzed human gene expression profiles from four renal glomeruli samples including two training datasets (GSE30528 and GSE1009) and two external validation datasets (GSE96804 and GSE47183). In our study, 120 common DEGs were identified in GSE30528 and GSE1009. GO and KEGG enrichment analyses were performed to explore the molecular mechanisms and signaling pathways of DN. The results revealed that the biological process of kidney development and urogenital system development was chiefly involved in the development of DN. The results of molecular functions showed that these DEGs may target actin binding, glycosaminoglycan binding, sulfur compound binding, and heparin binding to promote the progression of DN. And the KEGG enrichment analysis proved that these genes may affect DN through the PI3K-Akt signaling pathway, focal adhesion, complement and coagulation cascades, Rap1 signaling pathway, and regulation of actin cytoskeleton signaling pathways.

In order to further clarify the interaction relationship between these DEGs, we constructed the PPI network and identified the most important 10 hub genes including VEGFA, NPHS1, WT1, TJP1, CTGF, FYN, SYNPO, PODXL, TNNT2, and BMP2. Among these 10 hub genes, VEGFA, NPHS1, WT1, CTGF, SYNPO, PODXL, and TNNT2 were significantly downregulated in the DN renal glomeruli tissues compared with control samples, and the results could be verified by external validation datasets. Therefore, the reliability of downregulation of VEGFA, NPHS1, WT1, CTGF, SYNPO, PODXL, and TNNT2 may mediate the progression of DN can be verified at the molecular biological level. GFR is a well-recognized clinical indicator for evaluating renal function because the declining kidney function is typically assessed by a decline of GFR in clinical practice [16]. The clinical evidence from Nephroseq database showed that VEGFA, NPHS1, WT1, CTGF, SYNPO, and PODXL were positively correlated with GFR. Therefore, the credibility of VEGFA, NPHS1, WT1, CTGF, SYNPO, and PODXL as biomarkers for diagnosing and predicting the progression of DN can be proved at the clinical level. We can conclude that VEGFA, NPHS1, WT1, CTGF, SYNPO, and PODXL are not only reliable biomarkers for the diagnosis of DN but also predictive factors for the progression and prognosis of DN. Integrating these biomarkers and mechanisms with target drugs may accelerate the development of novel efficient drugs and treatment strategies. Therefore, we constructed the drug-gene interaction network by DGIDB online platform.

VEGFA encodes vascular endothelial growth factor A, and it is a member of platelet-derived growth factor superfamily [17]. VEGFA is highly expressed by glomerular podocytes and plays an important role in glomerular endothelial cell migration, differentiation, and survival [18]. Due to this, VEGFA can regulate glomerular structure and function which influence the outcome of DN. Therefore, proper VEGFA level is of great significance for maintaining normal glomerular function. Although some studies have found that there is a higher VEGFA level in diabetes, suggesting that the elevated VEGFA levels are pathogenic markers for diabetes [19]. However, VEGFA knock out diabetic mice has adverse consequences characterized by global sclerosis and death [18] which illustrate the opinion that the upregulation of VEGFA in diabetic kidneys may protect the microvasculature from injury and reduction of VEGFA in diabetes may be harmful. Another study directly provides evidence that the application of VEGF inhibitors could lead to glomerular injury [20]. A pharmaceutical experiment also proves that metformin increases the production of VEGFA in podocytes to reduce proteinuria through hypoxia-inducible factor-2*α*-VEGFA signaling pathway [21]. And a network pharmacology research shows that Huangqi Gegen decoction whose clinical efficacy has been widely confirmed [22] can bind with VEGFA to play the role of anti-inflammatory, antiapoptosis, antioxidation, and autophagy effects to improve renal function, thus delaying the development of DN [23]. All of these researches prove that VEGFA is beneficial to the establishment and maintenance of glomerular structure and function and provide a novel molecular mechanism for protecting renal function. Consistent with these studies, our research also demonstrated that VEGFA was significantly downregulated in the DN group. Therefore, we hold the opinion that elevated VEGFA may act as a compensatory protective mechanism in the progression of DN. But with the progress of disease, the expression level of VEGFA decreases dramatically, and decreased VEGFA indicates a seriously damaged endothelial system. We could believe that VEGFA plays a pivotal protective role in the pathogenesis of DN, and downregulated VEGFA is a reliable biomarker for DN [20]. To predict the potential effective therapy for DN associated with VEGFA, we applied the DGIDB database to determine therapeutic agents that might reverse the abnormally downregulated expression of VEGFA in DN. By checking drugs one-by-one in the network, we found that enalapril [24], sildenafil [25], and fenofibrate [26] positively regulate VEGFA. Enalapril is a commonly used drug to lower blood pressure and protect kidney function, fenofibrate is known as an important lipid-lowering drug, and sildenafil is an effective vasodilator. Since enalapril, sildenafil, and fenofibrate could target VEGFA and increase the expression levels of VEGFA, application of these drugs may reverse the abnormally downregulated VEGFA, thereby delaying the deterioration of renal function and providing new therapeutic targets for DN.

NPHS1 is primarily expressed in renal tissues and encodes nephrin protein [27] which is a key protein for the structural integrity and function of podocytes [28]. Therefore, NPHS1 is of great significance for maintaining glomerular filtration barrier in the kidney. Our study demonstrated that NPHS1 was significantly downregulated in the DN renal glomeruli tissues and positively correlated with GFR. Meanwhile, GO analysis showed that nephron development associated biological process was significantly enriched in DN. We can elucidate that the downregulation of NPHS1 may be involved in the occurrence and development of DN through destroying the integrity of glomerular filtration barrier. Therefore, reversal of the downregulated NPHS1 may protect renal podocytes to maintain the glomerular filtration barrier. In the drug-gene network analyses, we found that the target drug for NPHS1 was losartan, and this evidence was derived from an experimental study which proved that angiotensin II may cause a significant reduction of NPHS1 and losartan could restore angiotensin II-induced podocyte injury through Wnt/*β*-catenin axis [29]. A prospective multicenter randomized controlled trial (RCT) research also proved that NPHS1 variation is associated with the efficacy of losartan [30]. Therefore, the result that losartan can positively regulate the downregulated NPHS1 in DN patients may provide new evidence and mechanism for the usage of losartan in the treatment of DN.

Wilms' Tumor 1 (WT1) is mainly expressed in glomerular podocytes [31]. Therefore, WT1 plays a pivotal role in the formation of glomeruli and the normal function of podocytes [32]. Previous studies have proved that WT1 could ameliorate podocyte injury thus reducing urinary protein and serum creatinine and increasing GFR [33] via repression of the EZH2/*β*-catenin pathway in DN [34]. Our study further proved that WT1 was significantly downregulated in DN and positively correlated with GFR. Therefore, we could speculate the decreased protective effects of WT1 participant in the occurrence and progression of DN. Correspondingly, the reversion of the downregulated WT1 may delay the progression of DN. We found that halofuginone, deferoxamine, curcumin, and sirolimus had interactions with WT1 although the regulatory directions between them are yet unknown. But previous studies indicate that halofuginone could prevent extracellular matrix deposition and decrease oxidative stress [35], deferoxamine could suppress glomerular oxidative stress [36], and curcumin could alleviate podocyte epithelial-mesenchymal transition (EMT) [37], thereby suppressing the progression of DN. Sirolimus could reduce proteinuria and alleviate the early DN podocyte injury by inhibiting the activity of mTORC1 [38]. Therefore, halofuginone, deferoxamine, curcumin, and sirolimus are worthy of further exploration as potential therapeutic drug target for WT1 in the treatment of DN. It is noteworthy that there are various functions of WT1 [31]. So, the aberrations of WT1 are associated with different pathological variants of kidney diseases. Therefore, our findings are only generalizable to DN but not to other types of diseases.

A previous study has revealed that downregulation of connective tissue growth factor (CTGF), VEGFA, and WT1 was all related to a reduction of podocytes in DN [39]. Although our results also showed that CTGF was significantly downregulated in the DN group, there are many other studies show that overexpression of CTGF involved in the podocytes injury [40], and EMT in DN mice and CTGF antibody or inhibitors may protect podocytes from these injuries [41] and ameliorate DN [42, 43]. Therefore, the role of CTGF in the pathogenesis of DN requires to be further explored. Meanwhile, the application of CTGF-targeted drugs in the treatment of DN also needs further confirmation.

TNNT2 also known as troponin T2 is a subtype of the cardiac troponin family which plays a key role in the contraction of striated muscles [44]. Although it is generally believed that TNNT2 exists in cardiac muscle, the results of RNA expression profiles from two training datasets and two validation datasets all showed that TNNT2 was also expressed in human glomerular specimens and involved in the development of DN. Due to the fact that the loss of TNNT2 may lead to heart rhythm disorder and impaired cardiovascular function [45], so the downregulation of TNNT2 may symbolize the decline of cardiac function in DN patients. Therefore, downregulated TNNT2 is not only a symbol of deterioration of renal function but also represents the decline of cardiac function in DN patients. Hence, maintaining the normal level of TNNT2 may be helpful to delay the deterioration of renal function and improve the cardiac function in patients with DN. Purpurogallin, pyrogallol red, and chembl1601846 molecular compounds may target TNNT2 to achieve the above effects. Previous studies have proved that purpurogallin may play anti-inflammatory activities through inhibiting lipopolysaccharide- (LPS-) induced monocyte adhesion and migration and reducing the release of inflammatory mediators such as nuclear factor-*κ*B and tumor necrosis factor-*α* [46, 47]. Meanwhile, purpurogallin also has effects of antioxidant, antiplatelet, and antithrombotic activities [48], and it is a powerful protector of kidney [49] and an effective cardioprotector [50]. Combined with these effects of purpurogallin and our results from drug-gene interaction analyses, we think that purpurogallin may target TNNT2 to play a renal and cardioprotective effect for DN patients. Pyrogallol red and chembl1601846 were all chemical reagents, and their biological functions have not been sufficiently elucidated. Although information from the DGIDB database showed that pyrogallol red and chembl1601846 could target TNNT2, studies are still needed to confirm their biological functions and pharmacological effects. Furthermore, the efficiency of drugs or molecular compounds targeted to the key genes above still requires to be further validated by *in vitro*/*vivo* experiments and large-scale prospective cohort studies. Our *in silico* studies provide a possible clue. However, the present study exhibits some limitations. First, these solely *in silico* results require further validation by *in vitro*/*vivo* experimental studies. Second, we cannot know the specific CKD stage of the participants in these datasets. Therefore, the dynamic changes of these hub genes in the whole disease course need to be confirmed by clinical longitudinal cohort studies. Hence, collection of kidney specimens and more detailed clinical data from DN patients in a large cohort study is needed in the future to further validate our present findings. Meanwhile, high quality RCT researches are needed to confirm the effectiveness of these potential therapeutic drugs for DN.

Despite these limitations, we conclude that the present study provided a comprehensive bioinformatics analysis of DEGs and identified 10 hub genes that might be related to the progression of DN. We also revealed that key genes VEGFA, NPHS1, WT1, CTGF, SYNPO, PODXL, and TNNT2 were significantly downregulated in DN patients. VEGFA, NPHS1, WT1, CTGF, SYNPO, and PODXL were associated with the development and progression of DN at the molecular and clinical level. Various drugs provide new therapeutic targets for DN that enalapril, sildenafil, and fenofibrate may positively regulate VEGFA; and losartan positively regulates NPHS1. Halofuginone, deferoxamine, curcumin, and sirolimus are potential therapeutic drugs targeting WT1 in the treatment of DN. Purpurogallin may targetTNNT2 to delay the deterioration of renal function and improve the cardiac function in DN patients. To sum up, our study provides some promising biomarkers, mechanisms, and novel potential therapeutic targets for the scientific diagnosis and precise treatment of DN.

## 5. Conclusions

Our study provides new insights into the molecular mechanisms and targeted drugs of DN. VEGFA, NPHS1, WT1, CTGF, SYNPO, and PODXL are promising biomarkers for diagnosing and evaluating the progression of DN. The drug-gene interaction analyses provide a list of potential candidate drugs for the precise treatment of DN.

## Data Availability

The readers can access the data through GEO database.
